# Bilateral Adrenal Hemorrhage in Systemic Lupus Erythematosus and Antiphospholipid Syndrome: A Review of Mechanisms, Diagnosis, and Clinical Outcomes

**DOI:** 10.7759/cureus.98743

**Published:** 2025-12-08

**Authors:** Paola Pedraza Cruz, Dominique Digiacomo, Merina Varghese, Caleb T Spencer

**Affiliations:** 1 Internal Medicine, The University of Toledo College of Medicine and Life Sciences, Toledo, USA

**Keywords:** adrenal infarct, adrenal insuficciency, antiphospholipid antibody syndrome (aps), bilateral adrenal haemorrhage, hyper coagulable state, systemic lupus erythromatosus

## Abstract

Bilateral adrenal hemorrhage (BAH) is an uncommon but potentially fatal cause of adrenal insufficiency, particularly in patients with underlying hypercoagulable disorders. Autoimmune-mediated vasculitis, thrombosis, systemic lupus erythematosus (SLE), and antiphospholipid syndrome (APS) contribute to adrenal vascular compromise, yet literature on this association remains limited. We conducted a structured literature review using PubMed, focusing on articles in English published between 2000 and March 2025 that described BAH in the setting of SLE and/or APS. Search terms included “bilateral adrenal hemorrhage,” “systemic lupus erythematosus,” “adrenal insufficiency,” “antiphospholipid syndrome,” and related MeSH terms. Case reports, case series, and observational studies reporting BAH confirmed radiologically or histopathologically were included. The review identified autoimmune vasculitis, APS-related thrombosis, and hypercoagulability as major contributors to BAH. Clinical presentation is often nonspecific, with abdominal pain, hypotension, fever, and adrenal crisis as common features. Computed tomography (CT) remains the preferred initial imaging modality, while magnetic resonance imaging (MRI) offers superior sensitivity for early detection. Management requires prompt glucocorticoid and mineralocorticoid replacement, hemodynamic stabilization, and individualized anticoagulation strategies in APS-associated cases. Prognosis depends on timely diagnosis, correction of adrenal insufficiency, and control of underlying autoimmune activity. BAH in the context of SLE and APS remains a rare but life-threatening entity, and clinicians should maintain a high index of suspicion in autoimmune patients presenting with acute abdominal pain and hemodynamic instability. Early imaging, laboratory confirmation, and multidisciplinary management are essential for improving survival and long-term outcomes.

## Introduction and background

Bilateral adrenal hemorrhage (BAH) is a rare but potentially life-threatening cause of acute adrenal insufficiency, with a high risk of morbidity and mortality. If unrecognized or untreated, it can lead to severe hypotension, shock, and death. The clinical presentation can be subtle and nonspecific, often mimicking other acute conditions such as sepsis, which leads to delays in diagnosis. Timely recognition is critical, as the adrenal glands play a crucial role in hemodynamic stability, electrolyte regulation, and stress adaptation [1,2].

Autoimmune and hypercoagulable disorders are recognized risk factors, with systemic lupus erythematosus (SLE) and antiphospholipid syndrome (APS) among the most significant associations [3,4]. APS is characterized by the presence of antiphospholipid antibodies such as lupus anticoagulant, anticardiolipin, and anti-β2 glycoprotein I, which promote thrombosis in both arterial and venous circulations, including within the adrenal vasculature [5,6]. In SLE, APS may be secondary and confers an increased risk of thrombotic complications, with BAH resulting from adrenal vein thrombosis.

Although isolated reports describe BAH in APS or SLE, published data confirm its rarity. In APS, only around 0.4% of patients develop primary adrenal insufficiency (PAI) during follow-up, rising to 10-26% in catastrophic APS [2]. Similarly, in SLE, adrenal hemorrhage has been identified in only a handful of cases, with one cohort reporting six affected patients among several thousand, highlighting the rarity of BAH in autoimmune disease [6].

This review synthesizes existing literature to highlight shared mechanisms, diagnostic strategies, management, and outcomes in patients with BAH associated with underlying autoimmune hypercoagulability.

## Review

A targeted literature search was conducted using PubMed, MEDLINE, and Google Scholar to identify studies describing bilateral adrenal hemorrhage in the context of systemic lupus erythematosus or SLE/APS overlap. Search terms included combinations of keywords and Medical Subject Headings (MeSH) such as “adrenal hemorrhage,” “bilateral adrenal hemorrhage,” “systemic lupus erythematosus,” “antiphospholipid syndrome,” “adrenal insufficiency,” “hypercoagulable state,” “coagulopathy,” “adrenal crisis,” and imaging-related terms (e.g., “CT,” “MRI”), linked using Boolean operators (AND, OR, NOT). The initial PubMed search yielded ninety-nine results, which were screened by title and abstract, and full texts were reviewed when necessary. Studies not published in English were excluded. Studies were included if they specifically addressed adrenal hemorrhage in patients with SLE or SLE/APS overlap. Case reports, case series, and narrative reviews were prioritized, while articles unrelated to autoimmune-associated adrenal pathology were excluded. A total of thirty-four studies met the inclusion criteria. Given the rarity of the condition and the descriptive nature of available evidence, no formal data extraction tables or bias assessments were performed. Instead, key clinical features, proposed mechanisms, diagnostic strategies, and management principles were qualitatively synthesized to inform the review.

Pathophysiology

The adrenal glands have a rich arterial supply from the superior, middle, and inferior adrenal arteries but limited venous drainage through a single central vein, creating a high-flow but low outflow environment sometimes referred to as the “vascular dam” effect [7]. In stress or hypercoagulable states, this anatomy predisposes the glands to venous outflow obstruction, leading to a state in which intravascular pressure may exceed venous capacity, causing hemorrhagic infarction [8].

APS-related mechanisms include thrombosis within adrenal veins, microvascular occlusion from antiphospholipid antibody-mediated coagulation activation, and secondary infarction with hemorrhagic conversion. SLE-related mechanisms involve small-vessel vasculitis, immune complex deposition, and complement activation, which cause endothelial injury and further increase vascular fragility (Table 1) [6].

**Table 1:** Proposed Mechanisms of BAH in SLE/APS BAH: bilateral adrenal hemorrhage, SLE: systemic lupus erythematosus, APS: antiphospholipid syndrome.

Mechanism	Description
Adrenal vein thrombosis	APS-induced hypercoagulability causes venous occlusion and infarction
Vasculitis-induced fragility	SLE-mediated immune injury predisposes to rupture
Zona fasciculata lipid-rich reaction	Antiphospholipid antibody targeting promotes local coagulation
Stress-related venous congestion	Elevated catecholamines increase venous pressure

Clinical presentation and diagnosis

Symptoms of BAH are nonspecific, often presenting as abdominal or flank pain, hypotension, nausea, vomiting, or fever, which can mimic sepsis or adrenal crisis [9]. Computed tomography (CT) is the preferred initial imaging modality, although magnetic resonance imaging (MRI) detects early hemorrhage with greater sensitivity [10,11]. Laboratory evaluation should include serum cortisol and adrenocorticotropic hormone (ACTH), electrolytes, renin and aldosterone levels, and APS antibody panels such as lupus anticoagulant, anticardiolipin, and anti-beta 2 glycoprotein I [12]. If adrenal insufficiency is suspected, ideally blood samples for morning cortisol and ACTH should be drawn before empirical treatment with hydrocortisone (Table 2). Primary adrenal insufficiency should be suspected in patients with low morning serum cortisol levels and elevated ACTH levels, usually twofold the upper reference limit, pending confirmation with a synthetic ACTH stimulation test once the patient is stable [13,14]. Additionally, in these patients, high plasma renin concentration and low aldosterone concentration may confirm underlying mineralocorticoid deficiency. Electrolyte abnormalities associated with adrenal insufficiency may include hyponatremia, hyperkalemia, hypoglycemia, and hypercalcemia [14]. These laboratory disturbances, combined with low cortisol and elevated ACTH, provide essential diagnostic clues, especially when imaging findings are equivocal. 

**Table 2:** Diagnostic Approach to Suspected BAH in SLE/APS BAH: bilateral adrenal hemorrhage, SLE: systemic lupus erythematosus, APS: antiphospholipid syndrome, ACTH: adrenocorticotropic hormone.

Step	Evaluation
1	Assess hemodynamic status
2	Serum cortisol, ACTH, aldosterone
3	CT adrenal protocol
4	APS antibody testing
5	MRI if CT inconclusive or for subacute phase

Management

Initial treatment centers on glucocorticoid replacement with a stress dose of hydrocortisone 100 mg intravenous (IV) bolus followed by 50-100 mg IV every six to eight hours, then gradual taper. Mineralocorticoid replacement is provided concomitantly with fludrocortisone for persistent aldosterone deficiency. Volume resuscitation is critical and centers on giving isotonic saline to correct hypovolemia and hypotension, with vasopressor use required in the setting of shock [13,15]. However, management of BAH in patients with APS or SLE requires disease-specific considerations beyond standard adrenal crisis protocols. In these conditions, a hypercoagulable state poses a unique challenge regarding anticoagulation because patients remain at high thrombotic risk despite experiencing hemorrhage. Patients require lifelong anticoagulation to balance thrombosis versus bleeding risk. Warfarin (INR 2-3) is preferred, as direct oral anticoagulants (DOACs) are associated with higher recurrence rates, particularly in triple-positive APS [16,17]. Anticoagulation is typically resumed once the patient is hemodynamically stable and active bleeding has resolved, balancing hemorrhagic and thrombotic risks.

In SLE-related adrenal hemorrhage, immunosuppressive therapy is often required to control underlying vasculitis or associated complications such as lupus myocarditis. High-dose corticosteroids are first-line, with cyclophosphamide, rituximab, or intravenous immunoglobulin (IVIG) reserved for severe or refractory disease [18]. This combined endocrine, hematologic, and rheumatologic tailored approach distinguishes autoimmune-associated BAH from hemorrhage due to nonautoimmune etiologies and is critical for optimizing outcomes.

Prognosis and outcomes

Mortality is highest when the diagnosis is delayed or when concurrent severe autoimmune disease is present. Early initiation of steroid therapy significantly improves survival. Long-term, most APS/SLE-associated BAH survivors develop permanent adrenal insufficiency requiring lifelong glucocorticoid and mineralocorticoid replacement [19]. SLE with myocarditis markedly increases mortality risk due to combined adrenal and cardiac dysfunction [20].

## Conclusions

BAH in the setting of SLE and APS is an uncommon but life-threatening complication that demands early recognition and rapid intervention. Because its presentation can mimic other acute conditions, clinicians must maintain a high index of suspicion when patients with autoimmune disease develop abdominal pain, hypotension, or fever. Prompt imaging, timely initiation of glucocorticoid replacement, and hemodynamic support are essential to survival. Periodic reassessment of adrenal function with early-morning cortisol or ACTH stimulation testing is recommended every three to six months during the first two years after the initial diagnosis, followed by annual testing to evaluate potential recovery or progression to chronic adrenal insufficiency. Long-term management frequently requires lifelong hormone replacement and careful control of underlying autoimmune activity. Patients should be educated on adrenal crisis prevention and encouraged to seek prompt medical assistance if they notice hypotension, vomiting, or fever. A multidisciplinary approach is vital for optimizing outcomes, and increased awareness among physicians remains key to reducing delays in diagnosis and treatment.
